# Click Reactions as a Key Step for an Efficient and Selective Synthesis of d-Xylose-Based ILs

**DOI:** 10.3390/molecules180911512

**Published:** 2013-09-17

**Authors:** Nadège Ferlin, Sylvain Gatard, Albert Nguyen Van Nhien, Matthieu Courty, Sandrine Bouquillon

**Affiliations:** 1 Institut de Chimie Moléculaire de Reims, UMR CNRS 6229, Université de Reims Champagne-Ardenne, Boîte 44, B.P. 1039, Reims F-51687, France; E-Mails: nadege.ferlin@ipb.fr (N.F.); sylvain.gatard@univ-reims.fr (S.G.); 2 Laboratoire des Glucides FRE 3517, Université de Picardie Jules Verne, UFR des Sciences, 33 rue Saint Leu, Amiens Cedex 1 80039, France; E-Mail: albert.nguyen-van-nhien@u-picardie.fr; 3 Laboratoire de Réactivité et de Chimie des Solides UMR CNRS 7314, Université de Picardie Jules Verne, UFR des Sciences, 33 rue Saint Leu, Amiens Cedex 1 80039, France; E-Mail: matthieu.courty@u-picardie.fr

**Keywords:** ionic liquids, d-xylose, click chemistry

## Abstract

d-Xylose-based ionic liquids have been prepared from d-xylose following a five steps reaction sequence, the key step being a click cycloaddition. These ionic liquids (ILs) have been characterized through classical analytical methods (IR, NMR, mass spectroscopy, elemental analysis) and their stability constants, Tg and Tdec, were also determined. Considering their properties and their hydrophilicity, these compounds could be alternative solvents for chemical applications under mild conditions.

## 1. Introduction

**Figure 1:**
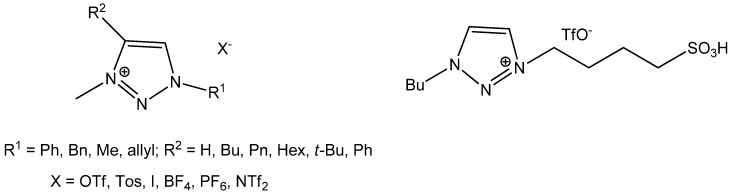
Triazolium based ionic liquids [13].

In the last two decades, ionic liquids (ILs) have attracted considerable attention due to their unique properties (non-flammability, good electrolytic properties, unique solubility, negligible vapor pressure, good thermal stability, *etc*.) [1,2,3]. Due to the increasing growth of their applications as alternative to volatile solvents in catalytic applications [1,4,5,6], biocatalysis [7,8], synthetic chemistry [9], electrochemistry [10,11,12,13,14], analytical applications [15,16,17,18,19], or for separations and extractions [20,21,22,23,24,25,26], the development of new IL structures is always being sought. Thanks to the click chemistry reaction, a large variety of 1,2,3-triazole structures can be obtained [27,28,29], but surprisingly, few ionic liquids derived from triazole have been reported (Figure 1) [30,31,32,33,34].

Carbohydrates are among the most abundant and low-cost natural sources of chiral materials and represent building blocks of choice for the formation of various compounds with a broad spectrum of applications [35]. The use of ILs as solvents for the transformation of carbohydrates was first reviewed by Linhardt in 2005 [36]. Next, ILs have been shown to exhibit excellent solubilizing properties, facilitating a wide range of chemical transformations, including acetylation, ortho-esterification, benzylidenation and glycosylation reactions of carbohydrates [36,37,38,39,40,41]. Recently, Afonso and Tran discussed respectively the application of ILs in carbohydrate dissolution [42] and the recent developments of ionic liquids in oligosaccharide synthesis [43]. Therefore, sugar-based chiral ionic liquids (CILs) could be used as solvent or catalyst in asymmetric synthesis [44,45,46,47,48] or as chiral phases in gas chromatography [49].

**Figure 2:**
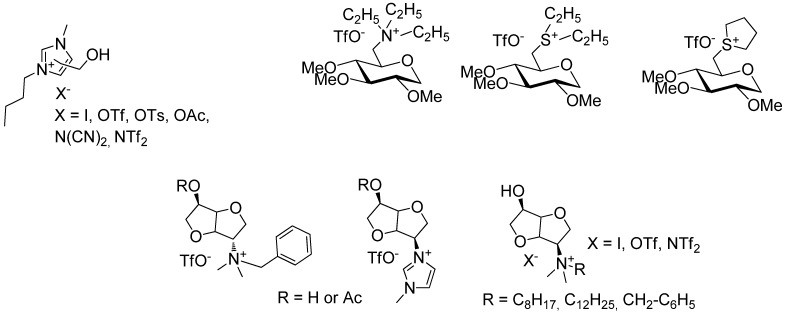
Carbohydrates-based ILs.

Only a few examples of carbohydrates-based ILs were reported in the literature [50,51,52,53,54,55,56,57] (Figure 2). First, in 2003 Dickenson *et al*. published the preparation of ILs derived from fructose as a promising solvent for implementing fully “green chemistry” methods [50]. Glucose was also used as starting material for the elaboration of either a new class of chiral solvents from low-cost natural sources [51] or multiphase particles for cosmetic applications [52]. Next, isomanide or isosorbide-based ILs were prepared as solvents for chiral discrimination or asymmetric organic reactions [53,54,55,56,57].

**Figure 3:**
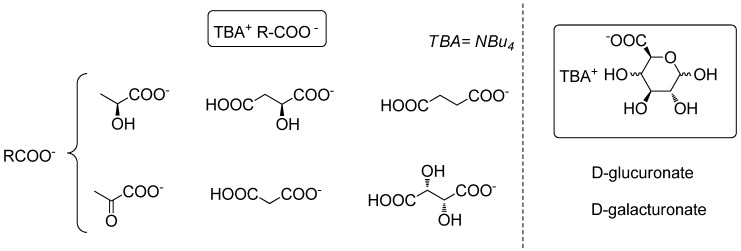
Biomass derived ILs.

For our part, we recently reported the preparation (and the use as solvent for catalysis) of biomass- derived ionic liquids from natural organic acids, among them osidic acids [58] (Figure 3). In this context, as we have been studying for many years the valuation of pentoses issued from hemicelluloses as surfactants [59,60,61,62,63,64] or glycodendrimers [65,66,67], we wish to report here a new way of valuation of these sugars as new ILs in which 1,2,3-triazolium salts [33,34,68,69,70,71,72,73,74,75,76] serve as the IL part and xyloside units are covalently tethered at the “4” position of the triazolium ring. To the best of our knowledge, no ionic liquid derived from d-xylose was previously described in the literature.

## 2. Results and Discussion

For the glycosylation step, treatment of peracetylated d-xylose with propargyl alcohol in the presence of BF^3^·Et^2^O was used to access the β-propargyl xyloside **1** [77]. This method was preferred because previous trials on d-xylose using the Fisher method [78] with para-toluenesulfonyl acid as catalyst led to a mixture of anomers which are could not be separated, even after acetylation.

**Scheme 1:**
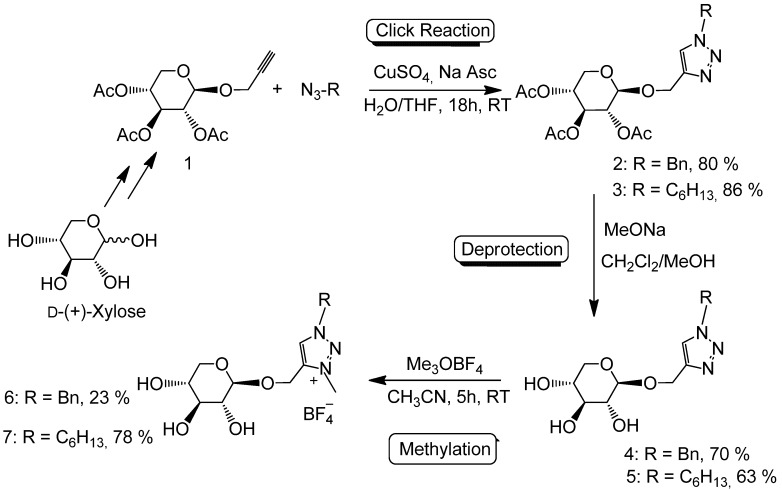
Synthesis of ILs **6** and **7**.

Cu^I^-“catalyzed” Huisgen 1,3-dipolar cycloaddition reaction of the modified alkynyl sugar with phenyl or hexyl azide, was carried out in the presence of an excess of Cu^I^ in a homogeneous THF/water mixture (Scheme 1). Several reactions were performed with catalytic and stoichiometric amounts of copper, but led to very poor yields, a part of the copper salt probably being involved in the complexation of the acetate groups. The propargyl xyloside/azide ratio was also optimized after several trials to afford good yields for the cycloaddition adducts.

The excess of Cu salt was removed as [Cu(NH^3^)^2^(H^2^O)^2^][SO^4^] by washing with an ammonia solution. Purification by precipitation with CH2Cl2/petroleum ether in order to remove the excess of sugar provided compounds **2** and **3** in good yields. The presence of signals at 7.42 ppm and 7.49 ppm for **2** and **3**, respectively, in their ^1^H-NMR spectrum, unambiguously proved the formation of the triazole ring. The composition of compounds **2** and **3** was further confirmed by ^13^C-NMR and elemental analysis. The acetylated benzyl and hexyl compounds **2** and **3** were then deprotected in the presence of sodium methanolate to give the corresponding derivatives **4** and **5** with free hydroxyl groups (Scheme 1). No signals were found for methyl groups or carbonyl carbons in the ^1^H- and ^13^C-NMR spectra, respectively. This set of derivatives was purified by precipitation.

In line with previous observations, trimethyloxonium tetrafluoroborate (Meerwein’s salt) proved to be a very powerful methylating agent (29 equivalents used as described [79]), affording benzyl and hexyl triazolium salts **6** and **7** in good isolated yields in 5 h at room temperature in dry MeCN (Scheme 1). Alternative reaction conditions applied to the hexyl derivative, using methyl iodide (20 equivalents) in dry MeCN under reflux gave improved yields (95%) but required longer reaction times (85 h). The new ILs **6** and **7** were highly soluble in water and in methanol and insoluble in diethyl ether, therefore their purification was done by precipitation of the crude products from MeOH/Et_2_O. The presence of signals around 4.32 ppm in their ^1^H-NMR spectrum and at 38.7 ppm in their ^13^C-NMR spectrum for the benzyl and hexyl derivatives, respectively, showed the quaternisation of the triazole ring.

**Figure 4:**
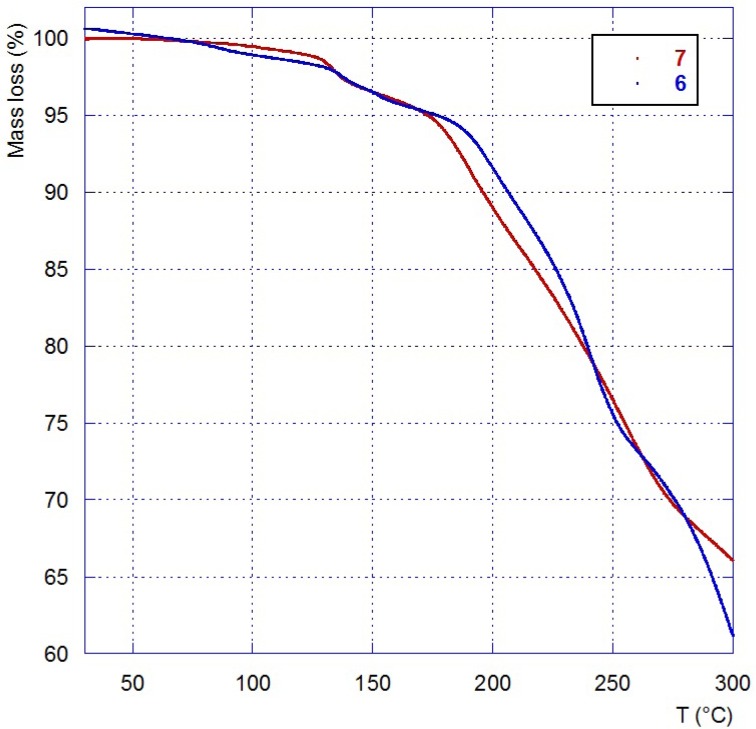
Thermogravimetric analysis of compounds **6** and **7**.

In addition of the IR, NMR, elemental analyses and mass spectroscopy, ILs **6** and **7** were characterized by DSC (Table 1) and TGA (Figure 4). Both compounds are stable until 120 °C and 150 °C, respectively, and showed a slight positive glass transition temperature (Tg). As previously described for tetrabutylammonium galacturonate and glucuronate [58], positive Tg and low decomposition temperature are observed what seems to be in relation with the presence of sugar moities. Considering these temperatures, **6** and **7** could be used only under mild conditions as solvents or chiral agents for chemical transformations or catalysis. 

**Table 1:** Glass transition and decomposition temperatures of ILs **6** and **7**.

IL	Tg (°C) ^a^	Tdec (°C) ^b^
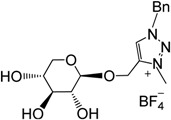	4	150
**6**		
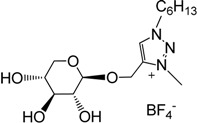	2.7	120
**7**		

^a^ Tg = Onset temperature measured at 10 K/min under argon; ^b^ Tdec = Onset temperature measured at 10 K/min under argon.

The thermal stability of **6** and 7 was determined by thermogravimetric analysis (TGA) under argon (Figure 4). The TG curve shows an initial weight loss of 1.33% and 0.76% of water respectively for **6** and **7** between room temperature and 110 °C followed by a second loss of water (3.20% and 3.01%). Such a noticeable mass loss corresponds to the hydroxyl groups. The thermal degradation (Tdec) occurring during the second step gives a loss of F (*m/z* = 19) fragments by mass spectrometry analysis originating from BF_4_^−^ decomposition.

## 3. Experimental

### 3.1. General Procedures

All reagents were commercially available and used as received. CH_2_Cl_2_ was dried over CaH_2_ and distilled under argon before use. CH_3_CN was dried using a Pure Solv solvent drying system over aluminum oxide under an argon atmosphere before use. ^1^H-NMR (250.1 MHz), ^13^C-NMR (62.9 MHz) and ^19^F-NMR (235.4 MHz) spectra were recorded on an AC 250 Bruker instrument in CDCl_3_ or MeOD with TMS as reference for ^1^H spectra and CDCl_3_ (δ 77.0) or MeOD (δ 49.9) for ^13^C spectra. IR spectra were recorded on a Nicolet AVATAR 320 FT-IR. C and H analyses were performed on a Perkin Elmer 2400 CHN equipment. Chromatographies were carried out on SDS Silica 60 (40–63 µm) or Silica 60 F_254_ (TLC plates). All experiments (MS and HRMS) were obtained on a hybrid tandem quadrupole/time-of-flight (Q-TOF) instrument, equipped with a pneumatically assisted electrospray (Z-spray) ion source (Micromass, Manchester, UK) operated in positive and negative mode. The electrospray potential was set to 3 kV in positive ion mode (flow of injection 5 μL/min.) and the extraction cone voltage was usually varied between 30 and 90 V. Optical rotations were measured on a Perkin Elmer 241 polarimeter. Thermogravimetric analyses coupled with a mass spectrometer were performed between 30 °C and 300 °C under a constant flow of dry argon (50 mL·min^−1^) using a Simultaneous Thermal Analyzer STA 449C Jupiter from Netzsch, and a heating rate of 10 K/min. The isothermal drift and sensitivity values are 0.6 µg/h and 0.1 µg, respectively. Alumina crucibles were loaded with 10–20 mg of sample. The DSC experiments were carried out on a Netzsch DSC 204F1 heat flux differential calorimeter at a heating rate of 10 K/min under a constant flow of dry argon (200 mL·min^−1^). Aluminum crucibles were loaded with 10–15 mg of sample.

### 3.2. Synthetic Procedures

#### 3.2.1. Preparation of 1-((1-Benzyl-1,2,3-triazol-4-yl)methoxy)2,3,4-tri-*O*-acetyl-β-d-xylopyranoside (**2**)


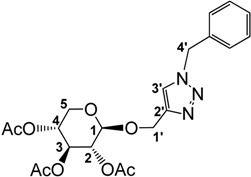


To a solution of β-propargyl xyloside **1** (2.08 g, 6.3 mmol) in a THF/water 1:1 (v:v) (20 mL) mixture were added benzyl azide (560 mg, 4.2 mmol), CuSO_4_·5H_2_O (4.2 g, 16.9 mmol), and sodium ascorbate (3.3 g, 16.9 mmol). The mixture was stirred at room temperature under an argon atmosphere for 18 h. The mixture was concentrated and CH_2_Cl_2_ was added. The organic layer was washed with aqueous ammonium hydroxide (0.8 M) until a colorless aqueous layer was obtained, then with water to neutrality. The organic phase was concentrated to dryness *in vacuo*. The crude product was dissolved in a minimum of CH_2_Cl_2_ and precipitated with an excess of petroleum ether. Compound **2** was obtained as a white solid in 80% yield (2.25 g). IR (KBr) ν cm^−1^: 2959, 2876, 1755, 1652, 1487, 1456, 1371, 1224, 1172, 1123, 1046. ^1^H-NMR (CDCl_3_) 1.82, 1.91, 1.96 (3 × s, 9H, C*H*_3Ac_), 3.37 (dd, *J* = 9 Hz, *J* = 11.7 Hz, 1H, *H*_5_), 4.11 (dd, *J* = 5 Hz, *J* = 11.7 Hz, 1H, *H*_5_), 4.61 (d, *J* = 5 Hz, 1H, *H*_1β_), 4.73–4.97 (overlap, 2H + 1H + 1H, *H*_1’_ + *H*_2_ + *H*_4_), 5.14 (t, *J* = 8.5 Hz, 1H, *H*_3_), 5.52 (s, 2H, *H*_4’_), 7.26–7.42 (overlapped, 5H, *H*_arom_), 7.42 (s, 1H, *H*_3’_). ^13^C-NMR (CDCl_3_) 20.4, 20.5, 20.6 (*CH*_3Ac_), 54.0 (*C*_1’_), 61.9, 62.3 (*C*_5_, *C*_4’_), 68.7, 70.5, 71.2 (*C*_2_, *C*_3_, *C*_4_), 99.6 (*C*_1β_), 122.6 (*C*_3’_), 128.1, 128.7, 129.0 (*C*H_arom_), 134.5 (*C*q_arom_), 144.5 (*C*_2’_), 169.3, 169.7, 169.8 (*C* = O_Ac_). Anal. Found (Calcd) for C_21_H_25_N_3_O_8_: C 56.39 (56.31), H 5.41 (5.62). [α]D^20^ = −71.9 (*c* 4.7, CHCl_3_).

#### 3.2.2. Preparation of 1-((1-Hexyl-1,2,3-triazol-4-yl)methoxy)2,3,4-tri-*O*-acetyl-β-d-xylopyranoside (**3**)


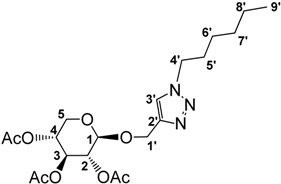


Same procedure as described for compound **2** was followed with a solution of β-propargyl xyloside **1** (3 g, 9.5 mmol) in a THF/water 1:1 (v:v) (20 mL) mixture, hexyl azide (809 mg, 6.4 mmol), CuSO_4_·5H_2_O (6.5 g, 26.0 mmol), and sodium ascorbate (5.1 g, 26.0 mmol). Compound **3** was obtained as a white solid in 86% yield (2.34 g). IR (KBr) ν cm^−1^: 2957, 2870, 1758, 1637, 1464, 1435, 1228, 1122, 1046. ^1^H-NMR (CDCl_3_) 0.85 (m, 3H, *H*_9’_), 1.30 (overlapped, 6H, *H*_6’_ + *H*_7’_ + *H*_8’_), 1.90 (m, 2H, *H*_5’_), 2.01, 2.03, 2.05 (3 × s, 9H, C*H*_3Ac_), 3.40 (dd, *J* = 9 Hz, *J* = 11.2 Hz, 1H, *H*5), 4.15 (dd, *J* = 5 Hz, *J* = 11.7 Hz, 1H, *H*_5_), 4.35 (t, *J* = 7.5 Hz, *H*_4’_), 4.64 (d, *J* = 6.5 Hz, 1H, *H*_1β_), 4.89–4.97 (overlapped, 2H + 1H + 1H, *H*_1’_ + *H*_2_ + *H*_4_), 5.17 (t, *J* = 10 Hz, 1H, *H*_3_), 7.49 (s, 1H, *H*_3’_). NMR ^13^C (62.9 MHz, CDCl_3_) 13.8 (*C*_9’_), 20.5 (*CH*_3Ac_), 22.9, 26.0, 30.1, 30.9 (*C*_5’_, *C*_6’_, *C*_7’_, *C*_8’_), 50.2 (*C*_1’_), 61.9, 62.4 (*C*_5_, *C*_4’_), 68.7, 70.6, 71.2 (*C*_2_, *C*_3_, *C*_4_), 99.6 (*C*_1β_), 122.3 (*C*_3’_), 144.0 (*C*_2’_), 169.3, 169.7, 169.8 (*C* = O_Ac_). Anal. Found (Calcd) for C_20_H_31_N_3_O_8_: C 54.34 (54.41), H 7.01 (7.08). [α]_D_^20^ = −67.0 (*c* 4.2, CHCl_3_).

#### 3.2.3. Preparation of 1-((1-Benzyl-1,2,3-triazol-4-yl)methoxy)β-d-xylopyranoside (**4**)


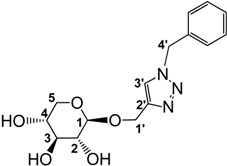


The acetylated compound **2** (170 mg, 0.38 mmol) was dissolved in CH_2_Cl_2_/MeOH 1:1 (v:v) (5 mL) under Ar and NaOMe (61.8 mg, 1.14 mmol) was then added. After stirring for 24 h at room temperature, the mixture was neutralized with Amberlite IR120 and filtered. The organic phase was concentrated to dryness *in vacuo*. The crude product was dissolved in a minimum of MeOH and precipitated with an excess of diethylether. Compound **4** was obtained as a white solid in 70% yield (m = 85 mg). ^1^H-NMR (CD_3_OD). 3.13–3.28 (overlapped, 1H + 1H + 1H, *H*_2_ + *H*_3_ + *H*_5_), 3.44 (m, 1H, *H*_4_), 3.82 (dd, *J* = 5 Hz, *J* = 11.2 Hz, *H*_5_), 4.26 (d, *J* = 7.5 Hz, 1H, *H*_1β_), 4.67 (d, *J* = 12.5 Hz, *H*_1’_), 4.87 (overlap, 3H + 1H, O*H* + *H*_1’_), 5.56 (s, 2H, *H*_4’_), 7.30 (m, 5H, *H*_arom_), 7.94 (s, 1H, *H*_3’_). ^13^C-NMR (CD_3_OD) 54.7 (*C*_1’_), 62.8 (*C*_4’_), 66.7 (*C*_5_), 70.9, 74.5, 77.3 (*C*_2_, *C*_3_, *C*_4_), 104.0 (*C*_1β_), 125.1 (*C*_3’_), 128.9, 129.4, 129.8 (*C*H_arom_), 136.5 (*C*q_arom_), 145.7 (*C*_2’_). Anal. Found (Calcd) for C_15_H_19_N_3_O_5_: C 55.97 (56.03), H 5.94 (5.92). [α]_D_^20^ = −36.7 (*c* 6.0, H_2_O).

#### 3.2.4. Preparation of 1-((1-Hexyl-1,2,3-triazol-4-yl)methoxy)β-d-xylopyranoside (**5**)


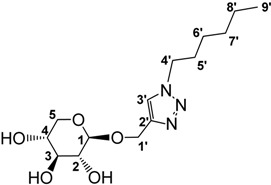


The same procedure as described for compound **4** was followed with compound **3** (2.34 g, 5.5 mmol) dissolved in CH_2_Cl_2_/MeOH 1:1 (v:v) (40 mL) and NaOMe (887 mg, 16.4 mmol). The compound **5** was obtained as a white solid in 63% yield (m = 1.09 g). ^1^H-NMR (CD_3_OD) 0.88 (m, 3H, *H*_9’_), 1.32 (overlapped, 6H, *H*_6’_ + *H*_7’_ + *H*_8’_), 1.88 (m, 2H, *H*_5’_), 3.18–3.31 (overlapped, 1H + 1H + 1H, *H*_2_ + *H*_3_ + *H*_5_), 3.48 (m, 1H, *H*_4_), 3.87 (dd, *J* = 5 Hz, *J* = 11.2 Hz, 1H, *H*_5_), 4.30 (d, *J* = 7.5 Hz, 1H, *H*_1β_), 4.38 (t, *J* = 7.5 Hz, 1H, *H*_4’_), 4.70 (d, *J* = 12.5 Hz, 1H, *H*_1’_), 4.87 (overlapped, 3H + 1H, O*H* + *H*_1’_), 7.97 (s, 1H, *H*_3’_). ^13^C-NMR (CD_3_OD) 13.9 (*C*_9’_), 22.0, 25.5, 29.7, 30.6 (*C*_5’_, *C*_6’_, *C*_7’_, *C*_8’_), 49.3 (*C*_1’_), 61.5 (*C*_4’_), 65.8 (*C*_5_), 69.6, 73.2, 76.6 (*C*_2_, *C*_3_, *C*_4_), 102.8 (*C*_1β_), 124.0 (*C*_3’_), 143.6 (*C*_2’_). Anal. Found (Calcd) for C_14_H_25_N_3_O_5_: C 53.28 (53.32), H 7.88 (7.99). [α]_D_^20^ = −40.0 (*c* 2.4, H_2_O).

#### 3.2.5. Preparation of 1-((1-Benzyl-3-methyl-1,2,3-triazol-4-yl)methoxy)β-d-xylopyranoside tetrafluoroborate (**6**)


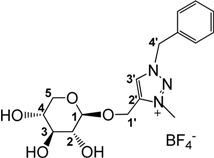


The corresponding triazole **4** (936 mg, 2.9 mmol) and Me_3_OBF_4_ (517 mg, 3.5 mmol) were stirred in dry acetonitrile (40 mL) for 5 h at room temperature. The reaction was quenched with MeOH (10 mL), and the solvent was removed under reduced pressure to give the crude product, which was in a minimum of MeOH and precipitated with excess of diethyl ether. Compound **6** was obtained as a white wax in 23% yield (m = 291 mg). IR: ν cm^−1^: 3363, 2891, 1737, 1635, 1589, 1456, 1348, 1286, 1244, 1155, 1035. ^1^H-NMR (CD3OD) 3.13–3.29 (overlapped, 1H + 1H + 1H, *H*_2_ + *H*_3_ + *H*_5_), 3.36 (m, 1H, *H*_4_), 3.85 (dd, *J* = 5 Hz, *J* = 11.2 Hz, *H*_5_), 4.35 (s, C*H*_3Tr_), 4.41 (d, *J* = 7.5 Hz, 1H, *H*_1β_), 4.87 (sl, 3H, O*H*), 5.05 (dd, *J* = 15 Hz, *J* = 20 Hz, 2H, *H*_1’_), 5.85 (s, 2H, *H*_4’_), 7.50 (m, 5H, *H*_arom_), 8.72 (s, 1H, *H*_3’_). ^13^C-NMR (CD_3_OD) 38.7 (*C*H_3Tr_), 58.0, 59.5 (*C*_1’_, *C*_4’_), 66.9 (*C*_5_), 70.8, 74.6, 77.5 (*C*_2_, *C*_3_, *C*_4_), 104.6 (*C*_1β_), 129.5 (*C*_3’_), 129.8, 130.0, 130.5 (*C*_Harom_), 133.4 (*C*_qarom_), 142.2 (*C*_2’_). ^19^F-NMR (CD_3_OD) 154.8 (s, B*F*4). Anal. Found (Calcd) for C_15_H_19_N_3_O_5_ + 1 H_2_O: C 43.96 (43.56), H 5.18 (5.48). [α]_D_^20^ = −13.1 (*c* 4.1 MeOH). HRMS calcd. for C_16_H_22_N_3_O_5_^+^: 336.1559, found 336.1555

#### 3.2.6. Preparation of 1-((1-Hexyl-3-methyl-1,2,3-triazol-4-yl)methoxy)β-d-xylopyranoside tetrafluoroborate (**7**)


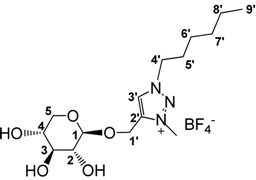


The same procedure as described for compound **6** was followed with the triazole **5** (900 mg, 2.8 mmol) and Me3OBF_4_ (506 mg, 3.4 mmol) in dry acetonitrile (40 mL). Compound **7** was obtained as as a white wax in 78% (m = 896 mg). IR: ν cm^−1^: 3392, 3140, 2956, 2929, 2872, 2494, 1589, 1460, 1356, 1323, 1286, 1247, 1038. ^1^H-NMR (250 MHz, CD_3_OD) 0.93 (m, 3H, *H*_9’_), 1.37 (overlap, 6H, *H*_6’_ + *H*_7’_ + *H*_8’_), 2.01 (m, 2H, *H*_5’_), 3.17–3.34 (overlap, 1H + 1H + 1H, *H*_2_ + *H*_3_ + *H*_5_), 3.49 (m, 1H, *H*_4_), 3.85 (dd, *J* = 5 Hz, *J* = 11.2 Hz, 1H, *H*_5_), 4.32 (s, 3H, C*H*_3Tr_), 4.37 (d, *J* = 7.5 Hz, 1H, *H*_1β_), 4.61 (t, *J* = 7.5 Hz, 1H, *H*_4’_), 4.87 (sl, 3H, O*H* sugar), 5.03 (dd, *J* = 7.5 Hz, *J* = 22.5 Hz, 2H, *H*_1’_), 8.71 (s, 1H, *H*_3’_). ^13^C-NMR (CD_3_OD) 14.3 (*C*_9’_), 23.4, 26.7, 30.1, 32.1 (*C*_5’_, *C*_6’_, *C*_7’_, *C*_8’_), 38.9 (*C*H_3Tr_), 54.9 (*C*_4’_), 59.6 (*C*_1’_), 67.0 (*C*_5_), 70.9, 74.5, 77.4 (*C*_2_, *C*_3_, *C*_4_), 104.8 (*C*_1β_), 130.7 (*C*_3’_), 142.0 (*C*_2’_). NMR ^19^F (235.4 MHz, CD_3_OD) 155.1 (s, B*F*4). Anal. Found (Calcd) for C_14_H_25_N_3_O_5_ + 1.5 H_2_O: C 40.11 (40.56), H 6.67 (7.03). [α]^20^_D_ = −20.4 (*c* 4.4 MeOH). HRMS calcd. for C_15_H_28_N_3_O_5_^+^: 330.2029, found 330.2033.

## 4. Conclusions

d-Xylose-based ILs have been prepared from d-xylose following an original pathway, the key step being a click cycloaddition. These ILs have been fully characterized and are hydrophilic. After determination of their ecotoxicity and their biodegradability in a near future, these solvents could be used as alternative solvents or chiral agents for synthesis or catalysis in water under mild conditions.

## References

[1] Wasserscheid P., Welton T. (2002). Ionic Liquids in Synthesis.

[2] Earle M.J., Esperança J.M.S.S., Gilea M.A., Lopes J.N.C., Rebelo L.P.N., Magee J.W., Seddon K.R., Widegren J.A. (2006). The distillation and volatility of ionic liquids. Nature.

[3] Couling D.J., Bernot R.J., Docherty K.M., Dixon J.K., Maginn E.J. (2006). Assessing the factors responsible for ionic liquid toxicity to aquatic organisms via quantitative structure-property relationship modeling. Green Chem..

[4] Olivier-Bourbigou H., Magna L., Morvan D. (2010). Ionic liquids and catalysis: Recent progress from knowledge to applications. Appl. Catal. A.

[5] Procuranti B., Myles L., Gathergood N., Connon S.J. (2009). Pyridinium ion catalysis of carbonyl protection reactions. Synthesis.

[6] Myles L., Gore R., Spulak M., Gathergood N., Connon S.J. (2010). Highly recyclable, imidazolium derived ionic liquids of low antimicrobial and antifungal toxicity: A new strategy for acid catalysis. Green Chem..

[7] Van Rantwijk F., Sheldon R.A. (2007). Biocatalysis in ionic liquids. Chem. Rev..

[8] Plaquevent J.-C., Levillain J., Guillen F., Malhiac C., Gaumont A.-C. (2008). Ionic liquids: New targets and media for α-amino acid and peptide chemistry. Chem. Rev..

[9] Welton T. (1999). Room-temperature ionic liquids. Solvents for synthesis and catalysis. Chem. Rev..

[10] Wei D., Ivaska A. (2008). Applications of ionic liquids in electrochemical sensors. Anal. Chim. Acta.

[11] Buzzeo M.C., Evans R.G., Compton R.G. (2004). Non-haloaluminate room-temperature ionic liquids in electrochemistry—A review. ChemPhysChem.

[12] Galinski M., Lewandowski A., Stepniak I. (2006). Ionic liquids as electrolytes. Electrochim. Acta.

[13] Ohno H. (2011). Electrochemical Aspects of Ionic Liquids.

[14] sterholm A., Damlin P., Kvarnström C., Ivaskaa A. (2011). Studying electronic transport in polyazulene-ionic liquid systems using infrared vibrational spectroscopy. Phys. Chem. Chem. Phys..

[15] Liu J.-F., Jiang G.-B., Jönsson J.A. (2005). Application of ionic liquids in analytical chemistry. TrAC Trends Anal. Chem..

[16] Baker G.A., Baker S.N., Pandey S., Bright F.V. (2005). An analytical view of ionic liquids. Analyst.

[17] Pandey S. (2006). Analytical applications of room-temperature ionic liquids: A review of recent efforts. Anal. Chim. Acta.

[18] Soukup-Hein R.J., Warnke M.M., Armstrong D.W. (2009). Ionic liquids in analytical chemistry. Annu. Rev. Anal. Chem..

[19] Sun P., Armstrong D.W. (2010). Ionic liquids in analytical chemistry. Anal. Chim. Acta.

[20] Baltus R.E., Counce R.M., Culbertson B.H., Luo H., DePaoli D.W., Dai S., Duckworth D.C. (2005). Examination of the potential of ionic liquids for gas separations. Sep. Sci. Technol..

[21] Han X., Armstrong D.W. (2007). Ionic liquids in separations. Acc. Chem. Res..

[22] Arce A., Earle M.J., Katdare S.P., Rodriguez H., Seddon K.R. (2008). Application of mutually immiscible ionic liquids to the separation of aromatic and aliphatic hydrocarbons by liquid extraction: A preliminary approach. Phys. Chem. Chem. Phys..

[23] Luis P., Garea A., Irabien A. (2009). Ionic liquids and membranes. J. Membr. Sci..

[24] Gorri D., Ruiz A., Ortiz A., Ortiz I. (2010). The use of ionic liquids as efficient extraction medium in the reactive separation of cycloolefines from cyclohexane. Chem. Eng. J..

[25] Meindersma G.W., Hansmeier A.R., de Haan A.B. (2010). Ionic liquids for aromatics extraction. Present status and future outlook. Ind. Eng. Chem. Res..

[26] Leskinen T., King A.W.T., Kilpeläinen I., Argyropoulos D.S. (2011). Fractionation of lignocellulosic materials with ionic liquids. Effect of mechanical treatment. Ind. Eng. Chem. Res..

[27] Huisgen R. (1961). 1,3-Dipolar cycloaddition. Proc. Chem. Soc..

[28] Himo F., Lovell T., Hilgraf R., Rostovtsev V.V., Noodleman L., Sharpless K.B., Fokin V.V. (2005). Copper(I)-catalyzed synthesis of azoles. DFT study predicts unprecedented reactivity and intermediates. J. Am. Chem. Soc..

[29] Rostovtsev V.V., Green L.G., Fokin V.V., Sharpless K.B. (2002). A stepwise huisgen cycloaddition process: Copper(I)-catalyzed regioselective “ligation” of azides and terminal alkynes. Angew. Chem. Int. Ed..

[30] Zekarias Yacob Z., Liebscher J., Handy S.T. (2011). 1,2,3-Triazolium Salts as a Versatile New Class of Ionic Liquids. “Ionic Liquids—Classes and Properties”.

[31] Jeong Y.-K., Kim D.-Y., Choi Y.-S., Ryu J.-S. (2011). Intramolecular hydroalkoxylation in Brønsted acidic ionic liquids and its application to the synthesis of (±)-centrolobine. Org. Biomol. Chem..

[32] Zhao F.Q., Xue L., Xing X.L., Hu R.Z., Zhou Z.M., Gao H.X., Yi J.H., Xu S.Y., Pei Q. (2011). Thermochemical properties and thermokinetic behavior of energetic triazole ionic salts. Sci. China Chem..

[33] Fletcher J.T., Keeney M.E., Walz S.E. (2010). 1-Allyl- and 1-Benzyl-3-methyl-1,2,3-triazolium salts via tandem click transformations. Synthesis.

[34] Jeong Y., Ryu J.-S. (2010). Synthesis of 1,3-Dialkyl-1,2,3-triazolium ionic liquids and their applications to the baylis-hillman reaction. J. Org. Chem..

[35] Hollingsworth R.I., Wang G. (2000). Toward a carbohydrate-based chemistry:  Progress in the development of general-purpose chiral synthons from carbohydrates. Chem. Rev..

[36] Murugesan S., Karst N., Islam T., Wiencek J.M., Linhardt R.J. (2003). Dialkyl imidazolium benzoates—Room temperature ionic liquids useful in the peracetylation and perbenzoylation of simple and sulfated saccharides. Synlett.

[37] Chiappe C., Marra A., Mele A. (2010). Synthesis and applications of ionic liquids derived from natural sugars. Top. Curr. Chem..

[38] Forsyth S.A., MacFarlane D.R., Thomson R.J., von Itzstein M. (2002). Rapid, clean, and mild *O*-acetylation of alcohols and carbohydrates in an ionic liquid. Chem. Commun..

[39] Rencurosi A., Lay L., Russo G., Caneva E., Poletti L. (2005). Glycosylation with trichloroacetimidates in ionic liquids: Influence of the reaction medium on the stereochemical outcome. J. Org. Chem..

[40] Murugesan S., Linhardt R.J. (2005). Ionic liquids in carbohydrate chemistry—Current trends and future directions. Curr. Org. Synth..

[41] Park T.J., Weiwer M., Yuan X.J., Baytas S.N., Munoz E.M., Murugesan R.J. (2007). Linhardt. Glycosylation in room temperature ionic liquid using unprotected and unactivated donors. Carbohydr. Res..

[42] Rosatella A.A., Frade R.F.M., Afonso C.A.M. (2011). Dissolution and transformation of carbohydrates in ionic liquids. Curr. Org. Synth..

[43] Galan C.M., Jones R.A., Tran A.-T. (2013). Recent development of ionic liquids in oligosaccharide synthesis. The sweet side of ionic liquids. Carbohydr. Res..

[44] Ogawa C., Kobayashi S. (2010). Catalytic Asymmetric Synthesis.

[45] Gaumont A.-C., Genisson Y., Guillen F., Plaquevent J.-C. (2011). De Pasteur aux liquides ioniques chiraux. Petite histoire de l’induction asymétrique promue par le solvant. Actualité Chimique.

[46] Gaumont A.-C., Genisson Y., Guillen F., Zgonnik V., Plaquevent J.-C., Gruttadauria M., Giaccalone F. (2011). Chiral ionic liquids for asymmetric reactions. Catalytic Methods in Asymmetric Synthesis.

[47] Chen X., Ying A., Kokorin A. (2011). Ionic liquids: Applications and perspectives. Ionic Liquids: Applications and Perspectives.

[48] Payagala T., Armstrong D.W. (2012). Chiral ionic liquids: A compendium of syntheses and applications. Chirality.

[49] Ding J., Welton T., Armstrong D.W. (2004). Chiral ionic liquids as stationary phases in gas chromatography. Anal. Chem..

[50] Handy S.T., Okello M., Dickenson G. (2003). Solvents from biorenewable sources: Ionic liquids based on fructose. Org. Lett..

[51] Poletti L., Chiappe C., Lay L., Pieraccini D., Polito L., Russo G. (2007). Glucose-derived ionic liquids: Exploring low-cost sources for novel chiral solvents. Green Chem..

[52] Quadir M., Mathonneau E. (2007). Cosmetic Composition Comprising Multiphasic Particles. U.S. Patent.

[53] Pereira M., Manuela A. (2012). Chiral ionic liquids from carbohydrates: Synthesis and properties. MiniRev. Org. Chem..

[54] Truong T.-K.-T., van Buu O.N., Aupoix A., Pegot B., Vo-Thanh G. (2012). Chiral ionic liquids derived from (−)-ephedrine and carbohydrates: Synthesis, properties and applications to asymmetric synthesis and catalysis. Curr. Org. Synth..

[55] Kumar V., Pei C., Olsen C.E., Schäffer S.J.C., Parmar V.S., Malhotra S.V. (2008). Novel carbohydrate-based chiral ammonium ionic liquids derived from isomannide. Tetrahedron Asymmetry.

[56] Nguyen Van Buu O., Aupoix A., Vo-Thanh G. (2009). Synthesis of novel chiral imidazolium-based ionic liquids derived from isosorbide and their applications in asymmetric aza Diels–Alder reaction. Tetrahedron.

[57] Nguyen Van Buu O., Aupoix A., Doan Thi Hong N., Vo-Thanh G. (2009). Chiral ionic liquids derived from isosorbide: Synthesis, properties and applications in asymmetric synthesis. New J. Chem..

[58] Ferlin N., Courty M., Gatard S., Spulak M., Quilty B., Beadham I., Ghavre M., Gathergood N., Bouquillon S. (2013). Biomass derived ionic liquids: Synthesis from natural organic acids, characterization, toxicity, biodegradation and use as solvents for catalytic hydrogenation processes. Tetrahedron.

[59] Estrine B., Bouquillon S., Hénin F., Muzart J. (2004). Telomerization of butadiene with l-arabinose and d-xylose in DMF: Selective formation of their monooctadienyl glycosides. Eur. J. Org. Chem..

[60] Estrine B., Bouquillon S., Hénin F., Muzart J. (2005). Telomerization of butadiene with pentoses in water: Selective etherifications. Green Chem..

[61] Hadad C., Damez C., Bouquillon S., Estrine B., Hénin F., Muzart J., Pezron I., Komunjer L. (2006). Neutral pentosides surfactants issued from the butadiene telomerization with pentoses: Preparation and amphiphilic properties. Carbohydr. Res..

[62] Damez C., Bouquillon S., Harakat D., Hénin F., Muzart J., Pezron I., Komunjer L. (2007). Alkenyl and alkenoyl amphiphilic derivatives of d-xylose and their surfactant properties. Carbohydr. Res..

[63] Deleu M., Damez C., Gatard S., Nott K., Paquot M., Bouquillon S. (2011). Synthesis and physico-chemical characterization of bolaamphiphiles derived from alkenyl d-xylosides. New J. Chem..

[64] Deleu M., Gatard S., Payen E., Lins L., Nott K., Flore C., Thomas R., Paquot M., Bouquillon S. (2012). d-xylose-based bolaamphiphiles: Synthesis and influence of the spacer nature on their interfacial and membrane properties. C. R. Chim..

[65] Hadad C., Majoral J.P., Muzart J., Caminade A.M., Bouquillon S. (2009). First phosphorous d-xylose derived glycodendrimers. Tetrahedron Lett..

[66] Camponovo J., Hadad C., Ruiz J., Cloutet E., Gatard S., Muzart J., Bouquillon S., Astruc D. (2009). “Click” glycodendrimers containing 27, 81 and 243 modified xylopyranoside termini. J. Org. Chem..

[67] Gatard S., Liang L., Salmon L., Ruiz J., Astruc D., Bouquillon S. (2011). Water-soluble glycodendrimers: Synthesis and use in hydrogenation catalytic process. Tetrahedron Lett..

[68] Hanelt S., Liebscher J. (2008). A novel and versatile access to task-specific ionic liquids based on 1,2,3-Triazolium salts. Synlett.

[69] Shah J., Kahn S.S., Blumenthal H., Liebscher J. (2009). 1,2,3-Triazolium-tagged prolines and their application in asymmetric aldol and Michael reactions. Synthesis.

[70] Nakamura T., Ogata K., Fukuzawa S.-I. (2010). Synthesis of dichlorobis(1,4-dimesityl-1H-1,2,3-triazol-5-ylidene)palladium [PdCl_2_(TMes)_2_] and its application to suzuki-miyaura coupling reaction. Chem. Lett..

[71] Nulwala H.B., Tang C.N., Kail B.W., Damodaran K., Kaur P., Wickramanayake S., Shi W., Luebke D.R. (2011). Probing the structure-property relationship of regioisomeric ionic liquids with click chemistry. Green Chem..

[72] Aizpurua J.M., Sagartzazu-Aizpurua M., Azcune I., Miranda J.I., Monasterio Z., García-Lecina E., Fratila R.M. (2011). “Click” synthesis of nonsymmetrical 4,4'-Bis(1,2,3-triazolium) salts. Synthesis.

[73] Ohmatsu K.O., Kiyokawa M., Ooi T. (2011). Chiral 1,2,3-Triazoliums as new cationic organic catalysts with anion-recognition ability: Application to asymmetric alkylation of oxindoles. J. Am. Chem. Soc..

[74] Khan S.S., Shah J., Liebscher J. (2011). Ionic-liquid tagged prolines as recyclable organocatalysts for enantioselective α-aminoxylations of carbonyl compounds. Tetrahedron.

[75] Yoshida Y., Takizawa S., Sasai H. (2012). Design and synthesis of spiro bis(1,2,3-triazolium) salts as chiral ionic liquids. Tetrahedron Asymmetry.

[76] Sanghi S., Willett E., Versek C., Tuominen M., Coughlin E.B. (2012). Physicochemical properties of 1,2,3-triazolium ionic liquids. RSC Adv..

[77] Mereyala H.B., Gurrala S.R. (1998). A highly diastereoselective, practical synthesis of allyl, propargyl 2,3,4,6-tetra-*O*-acetyl-β-d-gluco- or β-d-galactopyranosides and allyl, propargyl heptaacetyl-β-d-lactosides. Carbohydr. Res..

[78] Fischer E. (1895). Ueber die verbindungen der zucker mit den alkoholen und ketonen. Ber. Dtsch. Chem. Ges..

[79] Schulze B., Friebe C., Hager M.D., Günther W., Köhn U., Jahn B.O., Görls H., Schubert U.S. (2010). Anion complexation by triazolium “ligands”: Mono- and bis-tridentate complexes of sulfate. Org. Lett..

